# Feasibility of a Coordinated Human Papillomavirus (HPV) Vaccination Program between a Medical Clinic and a Community Pharmacy

**DOI:** 10.3390/pharmacy7030091

**Published:** 2019-07-14

**Authors:** William R. Doucette, Kelly Kent, Laura Seegmiller, Randal P. McDonough, William Evans

**Affiliations:** 1 Department of Pharmacy Practice & Science, University of Iowa, Iowa City, IA 52242, USA; 2 Towncrest Pharmacy, Iowa City, IA 52240, USA

**Keywords:** human papilloma virus, HPV vaccination, pharmacy, coordinated care

## Abstract

Human papillomavirus (HPV) vaccination coverage could be enhanced by community pharmacies working with medical clinics to coordinate completion of the HPV vaccination series. The objective for this study was to assess the feasibility of a coordinated model of HPV vaccine delivery in which a clinic gives the first dose and refers patients to a partnering community pharmacy to receive subsequent doses. A medical clinic-community pharmacy team was established in a Midwestern state to develop and operate a coordinated care model for HPV vaccinations. Under the coordinated model, the clinic identified patients needing HPV vaccination(s), administered the first dose and described the option to complete the vaccination series at the pharmacy. Interested patients then had an information sheet faxed and electronic prescriptions sent to the pharmacy. The pharmacy contacted the patients to schedule administration of 2nd and 3rd doses of the HPV vaccine. Over a 12-month period, 51 patients were referred to the pharmacy by the clinic. Of these, 23 patients received a total of 25 vaccinations. Clinic and pharmacy personnel mostly rated the coordinated program favorably. An initial study of a coordinated HPV vaccination program between a medical clinic and a community pharmacy supported patients getting HPV vaccinations.

## 1. Introduction

Human papillomavirus (HPV) is the most common sexually transmitted infection, with 14 million infections occurring in women and men in the United States (U.S.) annually [1]. HPV is responsible for approximately 33,700 cases of cancer each year, including virtually all cases of cervical cancer [2]. The infection is most common among young adults in their early teens through late twenties, in which it is transmitted sexually through contact with an infected person. The FDA approved the first vaccine for HPV in 2006. The HPV vaccine is recommended for adolescents beginning at 11 years of age, and consists of two doses administered over a course of six to twelve months, or three doses for those ages 15 and over [3]. In the four years following the recommendation, HPV infections in teenage girls decreased by 56% [4]. Despite the efficacy and safety of the vaccine, HPV vaccination rates have been substandard. In 2017, only 48.6% of teens reported HPV vaccination series completed, showing HPV vaccination rates are well below the *Healthy People 2020* national goal of 80% [5].

Innovative strategies should be considered in order to improve the vaccination rates among at-risk populations. In some countries, school-based approaches to delivering HPV vaccinations have been successful [6,7]. However, due to limited availability and concerns about payment, such school-based solutions are a challenge in the U.S. Pharmacists are healthcare professionals in a unique position to improve vaccine access and provide convenient administration of vaccines to patients, including the HPV vaccine. The National Vaccine Advisory Committee recommended using pharmacies to raise patient access to HPV vaccines [8]. Pharmacist involvement in vaccinations varies globally, with some countries allowing pharmacists to administer vaccines. These countries include Argentina, Australia, Philippines, South Africa, the UK and USA. These countries most commonly allow pharmacists to administer influenza vaccines, though other vaccines are allowed. In the US, pharmacists typically can administer influenza vaccines and other adult vaccines (e.g., for pneumococcus, or shingles) and travel vaccines. Much less common is ability of pharmacists to administer the “childhood vaccines” [9]. Pharmacists have historically offered several immunizations (e.g., influenza and pneumococcal) in the community setting and continually demonstrate their contribution to improved vaccination rates. A recent study assessed a 10 year span and compared vaccination rates between states that permitted pharmacy vaccination services and those that did not. The investigators found a significant increase in influenza vaccination rates among the states permitting pharmacist vaccinations [10]. Community pharmacies have collaborated with clinics to increase influenza vaccination rates [11]. Though such a coordinated model could be effective for raising HPV vaccination rates, no descriptions of this approach have been reported. It could be helpful to tap the potential of community pharmacists working in coordination with a clinic to address HPV vaccine series completion rates. The objective for this study was to assess the feasibility of a coordinated model of HPV vaccine delivery in which a clinic gives the first dose and refers patients to a partnering community pharmacy to receive subsequent doses.

## 2. Materials and Methods

This study recruited one primary care medical clinic and one community pharmacy to develop and evaluate a coordinated HPV vaccination program. To recruit a clinic-community pharmacy team, based on an author’s experience (W.R.D.) several progressive community pharmacies were approached, and interested pharmacists provided contact information for a clinician or clinic with which they wished to partner with for HPV vaccine delivery. An independent pharmacy located in a micropolitan area (population at least 10,000 but not more than 50,000) agreed to participate, along with a primary care medical clinic. The pharmacy provided traditional dispensing services, immunizations, adherence packaging services, medication management, and compounding services. The pharmacy employed a total of five pharmacist full-time equivalents (FTEs), a community pharmacy resident, and five technician FTEs. The two partners were located about two miles apart. The clinic is affiliated with a university health system, and employs about 20 providers. Once a clinic-pharmacy team was identified, the investigators contacted both providers and scheduled a 60 min face-to-face team building session for the pharmacy-clinic team members, including providers, the clinic manager, other clinic staff and pharmacy personnel. The team building session was facilitated by the research team, and consisted of an explanation of the project objectives with team discussion of roles and responsibilities in a coordinated HPV vaccination program.

In the USA, the credentialing of pharmacists to administer vaccinations varies by state. That is, each state’s governing/licensing body for pharmacists sets the requirements for pharmacists to be able to administer vaccines in that state. All 50 states have authorized pharmacists to administer vaccines at some level. Three states did not allow pharmacists to administer HPV vaccines (in 2015). In Iowa, the Board of Pharmacy allows pharmacists to administer HPV vaccines pursuant to a prescription order. It requires that a licensed pharmacist must complete an approved program on vaccine administration and complete training on basic life support for healthcare providers which includes hands-on training. There are not specific requirements for pharmacies other than providing proper storage for vaccines. Vaccinations typically are paid for by private insurance or through government program coverage, such as Vaccines for Children or Medicare for older patients. The preferred workflow of the coordinated program was determined by the clinic and pharmacy team members, including how patients would be identified, communication with the patients and between providers, reporting of immunizations in the state registry, and payment for the vaccinations. After the program was planned, practice changes and tools were made during a 4 month period, and then the coordinated program operated for a planned 12 month period. A brief online survey was used to collect feedback about the program from clinic and pharmacy staff. The survey asked about overall performance of the coordinated approach using a five-point scale (poor, fair, good, very good, excellent), challenges and benefits of the model using checklists, and an open-ended question about suggested improvements to the coordinated model. The Human Subjects Office at the University of Iowa approved this study.

The coordinated HPV vaccination program worked as follows. The clinic identified patients in need of HPV vaccination during its established patient care processes for preventive care for young patients. The first dose of the HPV series was administered within the participating clinic. To incorporate the coordinated program, all patients then were offered a choice of receiving the remainder of the HPV series vaccinations at the clinic or the pharmacy. For the patients who selected the pharmacy, the clinic then used electronic order sets developed with their IT personnel for second and third HPV vaccine doses and sent them to the participating pharmacy along with a patient information sheet, which contained demographic and contact information. The pharmacy was then responsible for working with the patient to schedule the remaining HPV vaccinations. During the administration visit at the pharmacy, patients were offered educational materials available in English and Spanish languages. The clinic and pharmacy served patients who spoke Spanish as their primary language. The pharmacist recorded the vaccinations in the Iowa Immunization Registry Information System (IRIS) and sent a clinical note in SOAP (Subjective, Objective, Assessment, Plan) format to the clinic partner. The pharmacy submitted billing to private insurers or to the Vaccines for Children (VFC) program for the doses administered there.

Prior to this study, the pharmacy had experience in administering vaccinations, including for influenza, pneumococcus, herpes zoster, and others. The pharmacy staff participated in a provider training session on the HPV vaccine provided by Merck. The patient education materials in English and Spanish were obtained from the CDC and other sources. During the study period, the pharmacy utilized a contact log to track communications with the patients, and used a texting service to remind patients of scheduled vaccinations.

The primary outcome variable for the coordinated HPV vaccination program was the number of HPV vaccinations delivered at the pharmacy. In addition, data were collected on the number of patients referred to the pharmacy by the clinic and the number of patients receiving an HPV vaccination at the pharmacy. Finally, changes in practice and tools developed for the program were collected from clinic and pharmacy staff.

## 3. Results

Both practices made changes in their operations to be able to work together within the coordinated HPV program. The clinic added electronic prescription order sets to be able to e-prescribe the second and third doses of the HPV vaccine to the pharmacy. In addition, the clinic developed a patient information sheet to fax to the pharmacy whenever they referred a patient for an HPV vaccine. This information included patient name, date of birth, address, insurance coverage and numbers, parent or guardian identification, and contact information. Finally, the clinic incorporated into their workflow a discussion with eligible patients about the option of completing the HPV vaccination series at the pharmacy. All providers participating at the clinic were physicians.

The pharmacy developed materials for patient communication, including a flyer about the HPV vaccination service in English and in Spanish, as well as a script for pharmacists to use when calling patients and/or their families about the HPV vaccination at the pharmacy. They also used “Oh Don’t Forget” texting to send patients a reminder before a scheduled vaccination. The pharmacy already routinely recorded vaccinations in Iowa’s Immunization Registry Information System (IRIS), and did so with the HPV vaccinations. For communicating with the clinic, the pharmacy utilized a clinical note template they already had in use that followed a SOAP format to inform the providers about each HPV vaccination administered. They also sent to the clinic a quarterly summary of the patients receiving an HPV vaccination. This report listed the patients receiving an HPV vaccination during that period, as well as the date of administration and the number of vaccine in the HPV series (e.g., 2nd or 3rd). Other practice changes made by the pharmacy included establishing themselves as a provider for the Vaccines for Children (VFC) program in Iowa, and developing a vaccination log to track communications with patients. The Vaccines for Children is a federal program that pays for vaccines for low income children.

During the 12 month study period, 51 patients were referred to the pharmacy by the clinic. Of these, 23 patients received a total of 25 vaccinations. All 23 patients completed their HPV series. Eighteen (78.3%) of the patients were female, while the mean age was 13.4 years (SD = 1.4). The insurance payments to the pharmacy were 13 (56.5%) commercial payers and 10 (43.5%) VFC. The other 28 patients either could not be reached by the pharmacy or declined to come to the pharmacy after being contacted. Surveys were received from five people at the clinic (four physicians and the clinic manager) and one from the pharmacy (pharmacist). Of the five respondents who rated the program’s performance, two rated it as excellent, and one each as very good, fair and poor. Reported difficulties in collaborating included having few interested patients, challenges with workflow, lack of staff time and some language barriers with the patients. Reported benefits to the coordinated program were that it made it easier for patients to complete the HPV vaccination series, increased opportunity for pharmacists and improved communication between the providers and pharmacists. When asked about changes to support such a team approach, having community pharmacist access to an electronic health record was the most common suggestion.

## 4. Discussion

The coordinated delivery of the HPV vaccine using clinic-pharmacy partnerships is a promising model for the improvement of HPV immunization rates through the use of alternative settings. As reported by the participating providers, some patients appreciated the convenience of getting an HPV vaccination at a pharmacy, which was open more hours than most clinics. This coordinated model improved patient access to HPV vaccinations, similar to the enhanced access provided for other vaccines, especially influenza. Previous work has shown that provider referrals are vital for pharmacies seeking to administer vaccines [12]. The coordinated model builds in provider referrals from the participating clinic. This study shows community pharmacies as a viable location for administration of HPV vaccinations. Future research should be conducted to investigate how a coordinated model could be implemented on a broader scale, perhaps involving a whole community instead of a single clinic-pharmacy team.

Positive provider recommendations to patients are key in getting them to agree to receive vaccinations, especially an HPV vaccine [13]. A strength of the coordinated program was that two voices were used to inform and encourage parents and patients to complete the HPV vaccination series. In addition to clinic personnel explaining the need for the HPV vaccine, a community pharmacist also discussed the HPV vaccination series with the patients and parents/caretakers. Pharmacists have been recognized as a trusted provider of medication and health information by patients [14], and can serve in a public health role. In addition, most pharmacists have completed training in providing immunizations, which supports their role in discussing HPV vaccinations with patients and/or their parents [15]. Together, primary care providers and pharmacists can help overcome parental hesitancy for HPV vaccinations [16]. This complementary communication can readily derive from a coordinated care approach being followed by a clinic-pharmacy partnership.

Another facilitator of this coordinated HPV program was that the clinic and the pharmacy exchanged patient information in a timely and usable manner. The patient information sheet sent by the clinic to the pharmacy allowed the pharmacy to receive necessary patient information, and provided successful patient hand-off communication [17]. The pharmacist was then able to contact the patient’s caretaker to discuss and schedule the next dose of the HPV vaccine. Similarly, the clinical note sent by the pharmacy to the clinic after vaccine administration let the clinic providers know that the patient had received another HPV vaccination. Another facilitator was the presence of HPV vaccination champions in both the clinic and the pharmacy. These people, a registered nurse and a pharmacist, helped assure that their respective organizations implemented the program and remained committed to it during the 12 month follow-up period.

As reported by the FIP in 2016, four countries allowed pharmacists to administer HPV vaccinations: Canada (some provinces), Portugal, the UK and USA [9]. It is hoped that the number of countries giving pharmacists authority to administer HPV vaccines will increase as more experience is gained with such services. A number of other countries allow pharmacists to administer influenza vaccines. As flu shots become more expected at pharmacies, it could be that other vaccines will be added to those that can be given by a pharmacist. Such changes likely would improve patient access to vaccines.

This study has several limitations. First is that only one clinic-community pharmacy team was studied. This limits the variability in the practice characteristics involved with the coordinated care model, including staff commitment, patient characteristics and community context. Having multiple clinics and multiple pharmacies using a coordinated care model likely would uncover more obstacles to be addressed in making the model work. This study showed that the coordinated care model involving a clinic and community pharmacy can be successful. However, future research with multiple clinic-pharmacy teams in a more rigorous design can address the limitation of just one team being studied. Another limitation is that no patient feedback was collected about their experiences with the coordinated care model. We do not know how satisfied they were with the vaccinations delivered at the pharmacy. As community pharmacists deliver more HPV vaccinations, patient feedback could be collected about their experiences with them.

## 5. Conclusions

An initial study of a coordinated HPV vaccination program between a medical clinic and a community pharmacy supported patients getting HPV vaccinations. More research is needed to extend and further test the effectiveness of this model.
